# Technical Considerations for Use of Oligonucleotide Solution API

**DOI:** 10.1089/nat.2020.0846

**Published:** 2020-08-06

**Authors:** Jale Muslehiddinoglu, Robert Simler, Malcolm L. Hill, Claudia Mueller, John H. A. Amery, Leigh Dixon, Anna Watson, Kirsten Storch, Cinzia Gazziola, Frank Gielen, Stefan Andreas Lange, Jeremy D. Prail, Doug P. Nesta

**Affiliations:** ^1^ Janssen, Beerse, Belgium.; ^2^ Biogen, Cambridge, Massachusetts, USA.; ^3^ GlaxoSmithKline, Stevenage, United Kingdom.; ^4^ F. Hoffmann-La Roche Ltd., Basel, Switzerland.; ^5^ Pfizer, Chesterfield, Missouri, USA.; ^6^ AstraZeneca, Macclesfield, United Kingdom.; ^7^ F. Hoffmann-La Roche Ltd., Basel, Switzerland.; ^8^ Janssen, Geel, Belgium.; ^9^ Sanofi, Frankfurt, Germany.; ^10^ GlaxoSmithKline, Worthing, United Kingdom.; ^11^ GlaxoSmithKline, King of Prussia, United Kingdom.

**Keywords:** oligonucleotides, lyophilization, solution API

## Abstract

The most common approach for the manufacture of oligonucleotides includes isolation of the active pharmaceutical ingredient (API) via lyophilization to provide a solid product, which is then dissolved to provide an aqueous formulation. It is well known from the development and manufacture of large molecules (“biologics”) that API production does not always require isolation of solid API before drug product formulation, and this article provides technical considerations for the analogous use of oligonucleotide API in solution. The primary factor considered is solution stability, and additional factors such as viscosity, concentration, end-to-end manufacturing, microbiological control, packaging, and storage are also discussed. The technical considerations discussed in this article will aid the careful evaluation of the relative advantages and disadvantages of solution versus powder API for a given oligonucleotide drug substance.

The European Pharma Oligonucleotide Consortium (EPOC) [1] is a collaboration between multiple pharma companies with the aim of sharing chemistry, manufacturing, and control (CMC) knowledge as well as strategies to enable harmonization of oligonucleotide development and commercialization.

The objective of the consortium is to publish science-based recommendations for the development of oligonucleotide therapeutics in a series of technical white papers, drawing on its collective subject matter expertise and complementing that in the literature. This public body of prior knowledge will serve as a reference for industry practice and help establish development principles for oligonucleotides. The consortium aims at being proactive and inclusive, and it anticipates initiating wider discussion on oligonucleotide CMC practice and policy, thus expediting access to these potentially life-changing medicines.

## Introduction

Oligonucleotides present a versatile therapeutic strategy with the potential to treat a wide range of diseases. Oligonucleotides have gained increased attention in the pharmaceutical industry, comprising such classes as antisense oligonucleotides, small/short interfering RNA, micro-RNA, immunostimulatory oligonucleotides, aptamers, and splice-switching oligonucleotides [2,3]. Before 2016, only three oligonucleotides had been approved for commercial therapeutic use. However, eight oligonucleotides have been approved in the past 4 years as tabulated in Table 1. The number of oligonucleotide programs in development in 2019, from research through Phase III, is listed as 609, compared with only 201 in 2010 [4]. Approved products are used in a range of therapies, including ophthalmologic indications, neuromuscular diseases, and adjuvants for vaccines. As they have become a greater percentage of the pharmaceutical industry's pipeline, increasing experience is leading to questioning pre-existing paradigms about the manufacturing process.

**Table 1.** Commercially Approved Oligonucleotide Therapeutics

Therapeutic	INN name	Class of oligonucleotide	Drug product container	Route of administration	Year approved
Vitravene^a^	Fomivirsen	Antisense	Vial	Intravitreal	1998
Macugen	pegaptanib sodium	RNA aptamer	Prefilled syringe	Intravitreal	2004
Kynamro	Mimopersen	Antisense	Prefilled syringe	Subcutaneous	2013
Defitelio	Defibrotide	Polydisperse mix of SS and DS	Vial	Intravenous	2016
Spinraza	nusinersen	MOE antisense	Vial	Intrathecal	2016
Exondys 51	eteplirsen	Morpholino	Vial	Intravenous	2016
Heplisav-B	Hepatitis B surface antigen	Immunosimulatory	Vial/prefilled syringe	Intramuscular	2017
Onpattro	patisiran	siRNA	Vial	Intravenous	2018
Tegsedi	inotersen	Gapmer antisense	Prefilled syringe	Subcutaneous	2018
Waylivra	volanesorsen	Gapmer antisense	Prefilled syringe	Subcutaneous	2019
Givlaari	givosiran	siRNA	Vial	Subcutaneous	2019

^a^ Withdrawn in Europe in 2002 and in the United States in 2006.

DS, double stranded; Inn, international nonproprietary number; MOE, methoxyethyl; siRNA, small/short interfering RNA; SS, single stranded.

Oligonucleotide active pharmaceutical ingredients (APIs) are manufactured by building the oligonucleotide chain on a solid support, adding a single nucleotide at a time. After the complete sequence has been generated, the oligonucleotide is cleaved from the solid support and subsequently purified, using chromatographic purification, usually reverse phase and/or ion exchange chromatography. If single stranded, the API in solution is then concentrated, desalted, and lyophilized to provide a powder API and if it is a duplex, after concentration and desalting of each strand, annealing is followed by further concentration and lyophilization.

Although oligonucleotide API is typically supplied as a powder, there may be advantages in supplying the API as a solution. This article summarizes the technical aspects that should be considered when deciding whether a solution could be beneficial to a particular program. The impact on both the API and drug product (DP) manufacturing processes, stability concerns, API container selection, impact to supply chain, and microbial/sterility control will be discussed.

The oligonucleotides considered in this article are chemically synthesized oligonucleotides, in particular based on the authors' experience with antisense oligonucleotides, including chemically modified versions to improve *in vivo* uptake or targeting, and nuclease stability. Although many of the considerations would, in principle, be applicable to oligonucleotides that are derived by a process that would be considered more aligned with biologic therapeutics, such as messenger RNA, these have not been specifically considered for this article.

In addition, it should be noted that for the purposes of the technical evaluation presented in this article, “solution API” refers to any presentation where the API has been solubilized in an aqueous medium. This encompasses solutions that may also include excipients that are present in the final DP formulation.

## Overview of Solution Versus Powder API

All current marketed oligonucleotide DPs are parenteral presentations and are manufactured as solutions in vials or pre-filled syringes. To enable manufacturing of the parenteral presentation, the powder API is typically dissolved in water during initial compounding operations during the DP manufacturing process. The last step in API manufacturing is the removal of water, and the first step of DP manufacturing is dissolution in water: Could the former be avoided, streamlining the end-to-end manufacturing process?

Powder API produced by lyophilization offers several advantages to the oligonucleotide DP manufacturing process. Powder API typically has excellent long-term stability on storage at −20°C, and in most cases refrigerated storage at 2°C–8°C may be acceptable. The powder is easy to ship to DP manufacturing sites. It also provides flexibility to the DP manufacturing process in that a range of final batch sizes and DP concentrations can be accommodated simply by varying how much API is dispensed during compounding. This can be especially important in case of patient weight-based dosing, where several different product strengths as well as different final fill volumes may be needed. Finally, microbial growth is less of a concern for powder API where the water content is significantly reduced.

In principle, as most oligonucleotide DPs are intended for parenteral administration, manufacturing processes using solution API should be more efficient. For example, moving the API solution directly into the sterilizing filtration for fill-finish eliminates the need for the lyophilization step of the API manufacturing process. Depending on the scale, the lyophilization step can easily be the bottleneck, from both batch output and cycle time perspectives. Such a bottleneck is accentuated in cases where the API batches need to be manufactured over a short time. In addition, lyophilization is an energy-intensive process.

A summary of key features to consider when assessing powder versus solution API is presented in Table 2, and the following sections further detail these. It should be noted that the focus of this article is to summarize these considerations, not to endorse one API presentation over another. The risk/benefit profile for the choice of API presentation is dependent on a number of factors, and the ultimate decision will be dependent on the needs of the product.

**Table 2.** Summary of Key Considerations in Assessing Solution and Powder Active Pharmaceutical Ingredient

Consideration	Solution API	Powder API
Stability	Oligonucleotides generally have enough stability for long-term storage in solution (>3 years either as a liquid or as frozen). Other stresses may be at higher risk in solution (ie, freeze/thaw, light, high temperature for some manufacturing processes).	Lyophilized oligonucleotides exhibit stability for >3 years in general under refrigerated and frozen conditions.
API manufacturing	Choice of concentration techniques dependent on final concentration needed for API. UF/DF may achieve up to 40–150 mg/mL concentration though the maximum may be sequence dependent. TFE or other evaporative process needed if high concentration is required.	UF/DF and lyophilization well established to isolate the API and transfer to the DP manufacturing process. Lyophilization step is time consuming (up to 5 days per batch).
Microbiologic considerations	Greater focus on microbial control, as aqueous environment may present a higher risk. Freezing is an option to prevent microbial growth. Controls have been historically well established for use in biologic manufacturing.	Powder API, particularly stored at −20°C, is less likely to promote microbial growth. Microbial growth is low risk.
Solution API packaging	Leachables are of greater concern with an aqueous environment, though acceptable leachable profiles have already been demonstrated with common containers. Supply chain implications of shipping larger masses of liquid, potentially under frozen conditions should be considered.	Powder API in drums stored at −20°C should have no leachable concerns and are easy to transport and store.
Integration with DP manufacturing	Greater efficiency by removing steps that are time consuming and potentially have critical/key parameters associated, that is, dissolution and compounding steps for fully formulated ready-to-fill API. Additional unit operations can be added (dilution, additional compounding) with other solution API presentations that still potentially provide manufacturing efficiency.	Requires dissolution, compounding, and dilution steps, thus adding more complexity to the DP manufacturing process.

API, active pharmaceutical ingredient; DP, drug product; TFE, thin film evaporation; UF/DF, ultrafiltration/diafiltration.

## Stability Considerations

Stability is a main driver when considering the feasibility of solution API. Sufficient stability at the chosen storage temperature is crucial and should be one of the first factors investigated. In general, oligonucleotides are very stable in solution at 2°C–8°C around neutral pH and with common excipients such as sodium chloride. Based on stability data at the intended long-term storage conditions as well as accelerated conditions, several marketed oligonucleotide DPs were approved in most markets with shelf-lives of 30 months at 2°C–8°C, for example, Spinraza^®^, Kynamro^®^. Thus, the stability of oligonucleotides supports that solution API can be a viable alternative to lyophilized API.

In general, stability testing has to show the impact of various environmental factors (such as temperature, light, humidity), as well as product-related factors (ie, stability at the required pH-range, interactions with container closure system, excipients or other APIs in case of fixed-dose combinations) on the quality of the API. The chosen storage conditions during stability testing as well as the evaluated storage lengths should cover the overall intended storage and shipment conditions to which the API is exposed within its commercial lifecycle. This also has to be accounted for during the end-to-end product development of solution API and liquid DP to obtain a commercially viable product. Although a powder API will, undoubtedly, be more resistant to degradation as a function of these stability variables, oligonucleotides in solution are generally stable enough to render concerns about stability.

Although structural differences present for different classes of oligonucleotides and some degradation pathways are not common, degradation products may be similar such as loss of nucleotides from the 3′- and/or 5′-termini, depurination, desulfurization (for phosphorothioate diester oligonucleotides), or oxidation [5–15]. In cases where the solution API product is not sufficiently stable during the overall intended storage time and distribution conditions at room temperature or 2°C–8°C, the product may also be stored frozen.

If frozen storage is to be considered for the API, it must be ensured that the product remains stable during the transition phases, that is, during the freezing and thawing unit operations, which are usually performed by evaluating one or several freeze/thaw cycles. Although it is the experience of the authors that the product quality of oligonucleotides is generally not impacted by freeze/thaw, it is important to confirm for each individual molecule in case exceptions arise. It should be noted that freezing and thawing are scale-dependent unit operations, therefore variability/heterogeneity in the freezing/thawing rates is not uncommon. For example, thawing may be passive, by allowing the solution API to thaw under ambient conditions, or active, where controlled thawing equipment is used to uniformly and predictably thaw the frozen API. During freezing, particularly at large scales, significant cryo-concentration of solution components may occur and thus result in considerable but localized formulation changes within cryovessels. For example, certain excipients, such as phosphate buffers, result in considerable pH shifts due to cryo-concentration and sequential precipitation of the acid and base component of the respective buffering system. Thus, the experimental set-up and analytical results for evaluation of freezing and thawing within a 300 L container may significantly differ from the one in a 5 L container and should therefore be considered within the experimental set-up for evaluation of the impact of freezing on critical quality attributes of the product [16,17].

During the formal stability testing for solution API (including frozen storage), representative small-scale containers may be used for economic reasons, even in instances where the final intended batch size may be several hundred liters in one container. In such cases, the contact materials of the small-scale containers should be representative of those intended for scale containers. Further, container contact surface area-to-liquid volume ratios as well as container headspace to liquid volume ratios within the small scale should be chosen such as to be the worst case.

In cases where solution API is considered a viable alternative to lyophilized API, the end-to-end product manufacturing approaches should also consider potential light sensitivity, since solution API is likely to be more photosensitive than a lyophilizate [18]. In case insufficient photostability according to procedures described within the ICH Q1B guideline is observed, measures during product storage and potentially even during manufacture to minimize light exposure should be considered. Light stability should be assessed early in development since sensitivity may be molecule specific.

Stability during the entire course of the solution API manufacturing process should also be considered with regards to processing temperatures. This may, in simple cases, be stability at room temperature during the process and any associated time out of refrigeration. In more complex cases, for certain manufacturing steps, for example, distillation, this may need to cover higher temperatures for short periods. If the manufacturing process requires these more extreme conditions to enable solution API, but the oligonucleotide is not stable under these conditions, solution API may not be possible for that molecule.

Another important aspect during product development of a solution API is viscosity. At high concentrations, the solution conditions deviate more and more from being ideal, resulting in increased oligonucleotide**–**oligonucleotide interactions due to molecular crowding phenomena. A similar principle is well described for highly concentrated protein formulations [19,20]. In case a high oligonucleotide concentration API is to be considered, the purification and formulation using ultrafiltration/diafiltration (UF/DF), and other manufacturing operations (see subsequent section) may become challenging or not even feasible to reach the required concentration due to increased viscosity.

The viscosity of a product is not only temperature and concentration dependent but may also vary with sequence, charge distribution/density, and type of oligonucleotide. Poecheim *et al.* measured a viscosity close to 120 mPas for a conjugated LNA molecule at 500 mg/mL, whereas a nonconjugated locked nucleic acid (LNA) molecule showed only 80 mPas viscosity at the same conditions [20]. Addition of viscosity reducing excipients to the formulation may be considered [21,22].

Ensuring solution API stability during shipping is key, even for early stage development programs. Such studies comprise evaluation of commonly encountered shipping stresses such as temperature excursions and agitation stress. Oligonucleotides do not generally exhibit sensitivities to shear stress and transient temperature excursions, which can be problematic for biologics during shipping qualification.

## Solution API Manufacturing: End-to-End Process Considerations

**FIG. 1.**
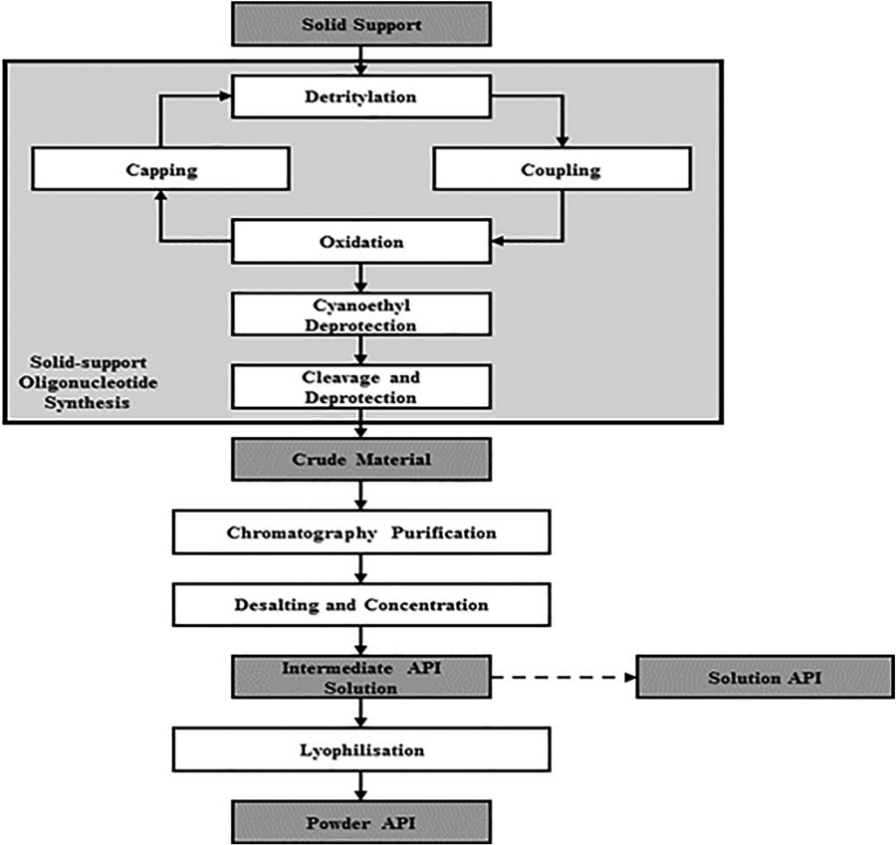
The typical unit operations for the manufacture of oligonucleotide API. API, active pharmaceutical ingredient.

The practical aspects of the manufacture of solution API, including the nature of the solution, how it is processed, and the fit within the proposed supply chain are considered in this section. The typical unit operations for the manufacture of oligonucleotide API are shown in Fig. 1, and this section discusses alternative processing of the API solution. The arguments and data presented refer to single-stranded oligonucleotides and suggest that the approach would be applicable to similar oligonucleotides manufactured by using solid supported synthesis with chromatographic purification. The arguments presented may be used as a starting point for other classes of oligonucleotides, and each case should be supported with data.

The first step toward an oligonucleotide API solution process is determining the required concentration of the API solution. Where allowing for the addition of any formulation materials such as buffers and the use of line washes in the compounding process, the target API concentration will need to be higher than the final DP concentration. Depending on the required concentration, achieving the ideal case of the target DP concentration “ready to fill” (and in the formulation required for DP manufacture) during API manufacture may require additional unit operations to be included. The API solution will typically be concentrated using a physical process such as membrane filtration and/or distillation. Distillation is likely to be required where the DP is at a high concentration, for example, >150 mg/mL, but can be lower depending on the nature of the oligonucleotide, as it is unlikely that membrane filtration would be practicable. When considering distillation approaches, it is essential that there is adequate solution stability to cover the expected processing temperature, time ranges, and reasonable excursions that could occur.

Where API is isolated as solid by precipitation, solution API could be generated by dissolution of the solid after the last precipitation. However, as organic antisolvents are used in the precipitations, control of solvents in API without drying of the final solid is likely to be considered.

There are benefits and drawbacks to manufacturing API in solution at high concentrations. The benefits include more flexibility during the DP process in the dose ranges achievable during manufacturing and lower transport costs due to transporting lower masses of water to the DP manufacturing site. The disadvantages include a potentially higher viscosity solution that is more difficult to handle, process, and clean; additional unit operations may be required in the API process to achieve the higher concentrations required, and the potential for higher yield losses to residual solution in equipment lines and API packaging.

## Methods for Concentration of the Oligonucleotide API Solution

The unit operations chosen for the manufacture of a particular solution API will depend on the nature of the oligonucleotide, the concentration required for DP manufacture, and the resulting viscosity. As organic solvents are used in the synthesis steps and in some instances during chromatographic purification, there is the possibility that these may be present in the API solution before concentration. Where organic solvents are present in the unfinished API solution after purification, sufficient removal of these during the concentration step will need to be demonstrated.

### Membrane procedures

UF/DF uses tangential flow filtration membranes with a pore size of 0.001–0.1 μm. UF membranes retain molecules that are larger than the pores of the membrane in the retentate solution; whereas smaller molecules such as salts, solvents, and water pass through the membrane into the permeate solution. The filtration properties of the membranes are described via the Molecular Weight Cut-Off (MWCO). UF is a concentration process whereas DF removes, replaces, or reduces small molecules, such as salts, in the API solution [23].

A benefit of UF/DF in the API manufacturing process shown in Fig. 1 is that one unit operation serves two purposes, that is, removal of salts introduced during the chromatographic purification step, and concentration of the solution. In addition, salt exchange can be carried out in this equipment, if required. Further, UF/DF can potentially purge small-molecule impurities. There is also an opportunity to simplify the overall oligonucleotide DP process by DF directly into the DP buffer.

The potential downsides to UF/DF should be considered. Large volumes of water are required for DF. The maximum concentration achievable by UF may be limited by gel layer formation on the membrane (fouling). This gel layer will be dependent on the nature of the oligonucleotide and excipients present during the UF/DF operation. However, higher concentrations are likely to promote the formation of the layer. This will result in a slow transfer of water through the membrane, lengthening, and potentially preventing from reaching the desired concentration, thus limiting the capability of the process.

The maximum concentration possible by using membrane filtration will depend on the API solution components, the scale, the minimum volume of the feed and retentate tanks, and the MWCO of the membrane. Concentrations of 40–100 mg/mL are regularly achieved and it may be possible to achieve higher concentrations by increasing the MWCO of the membrane; however, this could lead to yield loss through the membrane. Further work would be required to understand whether it is possible to use UF to exceed the concentrations typical for subcutaneous DPs and compare the practicability of this with distillation procedures.

### Distillation procedures

Thin film evaporation (TFE), or wiped film evaporation [24–28], is a distillation process where the solution flows through cylindrical equipment and is wiped around the internal wall by blades or rollers to form a thin film. A jacket is used to heat the walls of the equipment and the process takes place at reduced pressure, causing water to evaporate from the oligonucleotide solution as it travels through the evaporator.

The main benefits of TFE include the short residence time at elevated temperatures compared with still/pot distillation, which reduces the risk of thermal degradation. In addition, it is likely that TFE will achieve higher concentrations of API solution compared with UF/DF, as more viscous liquids are less likely to foul the evaporator. The process can be scaled by using equipment with increased evaporation area, thus maintaining practicable processing times as the process is scaled up. TFE is amenable to process modeling [25,27] and there is the potential for continuous manufacture. A downside of TFE is that salts cannot be removed, and it is therefore likely to be introduced as an additional API process step after UF/DF.

The maximum concentration of API solution achieved during manufacture by the authors to date is 160 mg ASO/g solution. It may be possible to achieve higher concentrations; however, this has not yet been proven. For TFE processes, cycle times and total evaporation times will be dependent on batch size, concentration factor, and evaporation area. In general, this can be completed in a time frame of a few hours.

Rotary evaporation is a distillation process where the solution to be concentrated is placed in a spherical vessel, rotated, and heated at reduced pressure. As with TFE, this process can achieve high concentrations, is unlikely to be foul, and is additional to UF/DF as it does not remove salts. Crucially, rotary evaporation requires a longer residence time at elevated temperatures compared with TFE, increasing the potential for degradation of the oligo API. This process is also difficult to scale up due to equipment availability and the increase in residence times with scale. The maximum concentration achievable by rotary evaporation is likely to be similar to TFE, but increased degradation of the API in achieving this is a risk requiring careful consideration.

For scale-up, vacuum still distillation may be considered, and the temperatures and times required should be evaluated alongside the feasibility of routinely achieving the low vacuum required to remove water without significant degradation of the oligonucleotide.

## Microbiologic Consideration

The chemical synthesis of an oligonucleotide is analogous to that of small-molecule manufacturing from microbiologic perspective since it is run in solvents (Fig. 1). However, once the oligonucleotide is in aqueous solution either after chromatographic purification or UF/DF, it is important to demonstrate low microbial contamination (bioburden) during the aqueous parts of API manufacturing and for the final release. Experience gained during the manufacture of biopharmaceuticals can be employed with the acknowledgement that the risk profile for microbiological contamination is different for oligonucleotides. Oligonucleotides are manufactured via solid-supported synthesis followed by chromatographic purification steps that use organic solvents and only the last step is in aqueous solutions. On the contrary, biologics are manufactured by using conditions that highly favor microbial growth such as aqueous solutions, nutrients, and neutral pH. Bioburden is a measure of the quantity of viable micro-organisms present in a sample. European Pharmacopoeia (EP Pharm Eur.) 2.6.12 Microbial Examination of Non-sterile Products: Total Viable Aerobic Count and USP<61> Microbiological Examination of Nonsterile Products: Microbial Enumeration Tests are typically followed to evaluate bioburden levels. The levels of endotoxins must also be controlled in oligonucleotide solutions. Guidance on methodology is available in EP 2.6.14 Bacterial Endotoxins and USP<85> Bacterial Endotoxins. Similar to microbial contamination, there is a different risk profile for endotoxins in oligonucleotide solutions compared with biologics. Further, in the process for powder API, the API solution may be passed through a 0.22-μm filter immediately before loading in the lyophilizer as an additional control. This bioburden reduction filtration is also recommended for solution API after final concentration and before API container filling. During the drug substance manufacturing process, levels <1 CFU/mL demonstrate microbial control if bioburden reduction is performed before the sterile filtration step in DP manufacturing but higher levels may be justifiable on a case-by-case basis. During the DP manufacturing process, levels of ≤10 CFU/100 mL meet regulatory expectations for the pre-sterile filtration of formulated bulk DP [29]. However, a pre-sterile filtration level of ≤1 CFU/10 mL might be warranted as it is mathematically comparable to the regulatory expected limit (≤10 CFU/100 mL), but allows for a smaller volume of material to be tested.

Historically, for biologics there has been some regulatory expectation that bioburden is controlled during API storage. This can be accomplished by performing bioburden analysis or confirming container closure integrity. Bioburden control may be important for certain storage conditions and container closures. Frozen conditions are not conducive to microbial growth in API, and pre-sterilized modern drug substance primary containers are well designed to maintain closure integrity over the range of conditions anticipated during storage and shipping. For API stored as a non-frozen liquid, the container closure and facility environmental controls would be assessed as the primary factors to prevent microbial intrusion. Both bioburden testing and container closure integrity can be considered viable options for demonstrating microbial control on stability. It is important for frozen API solution to assess whether the freezing process may compromise the container components.

## Packaging and Storing the Solution of API

As noted in the previous section on stability, when choosing a system for packaging and storage of the solution API, demonstrating the stability of the molecule at the recommended storage temperature (including freeze**–**thaw stability in case the liquid API is to be stored frozen) is critical. The suitability of the container system for the batch size and recommended storage conditions must also be considered, and a range of multiple or single use systems are available. Sterile single-use systems include bottles and bag systems. Common contact materials for bottles include polytetrafluoroethylene, polycarbonate, or polyethylene terephthalate glycol; whereas ethyl vinyl acetate, high-density polyethylene, or ultra-low-density polyethylene are often used for bags. For any contact material employed for solution API storage, material compatibility as well as extractables and leachable studies will have to be performed in a phase-appropriate manner. In addition, oxygen transmission rates through the container are important to characterize whether ingress can adversely affect product quality over time. Batch size will also be a key consideration for the API packaging. For instance, since bags can generally accommodate more volume than bottles, larger batch sizes may be better accommodated with bags so as to reduce the number of containers required for storage and therefore utilize less storage space.

Some polymers used for single-use storage systems may become brittle at low temperatures (below 2°C–8°C). This may lead to increased breakage of the bottles or bags and the associated tubing and connectors, and this must be accounted for where frozen storage is proposed. To prevent or minimize bag leakage/breakage during freezing and thawing operations, systems using supportive shells were developed to protect bags, connectors, and tubing from mechanical impact during handling. The shells should be considered for evaluation in case of frozen API storage in single-use bags to mitigate the risk of leakage. Some construction materials for single-use bottles, for example, PTFE, can be stored down to −80°C without becoming brittle. Where shipping on dry ice at −80°C is proposed, it should be noted that plastics usually show higher gas permeability than stainless steel systems and potential carbon dioxide ingress may result in possible pH shifts during shipping.

Multiple-use systems for solution API storage mainly comprise cryovessels or cans made of stainless steel alloys, such as 316 L or Hastelloy^®^ (available up to 300 L). Since cryovessels offer the advantage of actively cooled and heated freeze and thaw processes, freezing-rate heterogeneity even at large scales can be better controlled.

Supply chain implications are also critical when selecting the packaging for the API and evaluating whether solution API is a viable alternative for lyophilized powder API. In general, shipping and storage complexity increases as containers become bigger and temperatures become lower. Thus, the shipping and storage of a solution API will be more cumbersome than that of a powder API. The footprint of powder API containers is usually very small, making storage and shipping relatively facile, even at temperatures at −20°C and below. Solution API batches could be tens to hundreds of liters in size depending on the concentration chosen for the API. Ensuring that shipping logistics, storage space, and facility controls are met during not only storage but also during temperature ramps required for DP manufacturing is sufficiently more burdensome at these batch sizes. This becomes even more complex should freezing be required, as shipping large volumes of frozen material can be logistically difficult. Having appropriate controls and equipment in place to ensure freezing and thawing are performed within parameters determined from previously described freeze/thaw studies further complicates supply chain management.

Because of the supply chain logistics, attention should be paid to picking the optimal API configuration. For example, an API batch of 2 kg isolated as a 50 mg/mL solution will have a batch volume of 40 L. Storage of this volume in a single bag is feasible should the product demand warrant a DP batch of this size. However, sub-division of the API into smaller containers can provide several advantages such as flexibility in tailoring batch size to product demand and can also be considered in conjunction with other factors presented here for optimal API storage containers.

Finally, where long-term storage of solution API is planned, consideration should be given to the ease of sampling for retest from the API container. The key considerations are the time to warm to ambient temperature, the potential impact of warming on quality, and ensuring that the sampled solution is homogeneous; for example, demonstration of the homogeneity of a liquid thawed from frozen in an opaque hard-walled container. A suitable sampling method will also be required to ensure chemical and microbiological integrity of the solution; for example, a bag or hard-walled container system with specialized sampling ports will reduce the risk of contamination.

## General DP Manufacturing Process Considerations for Solution API

**FIG. 2.**
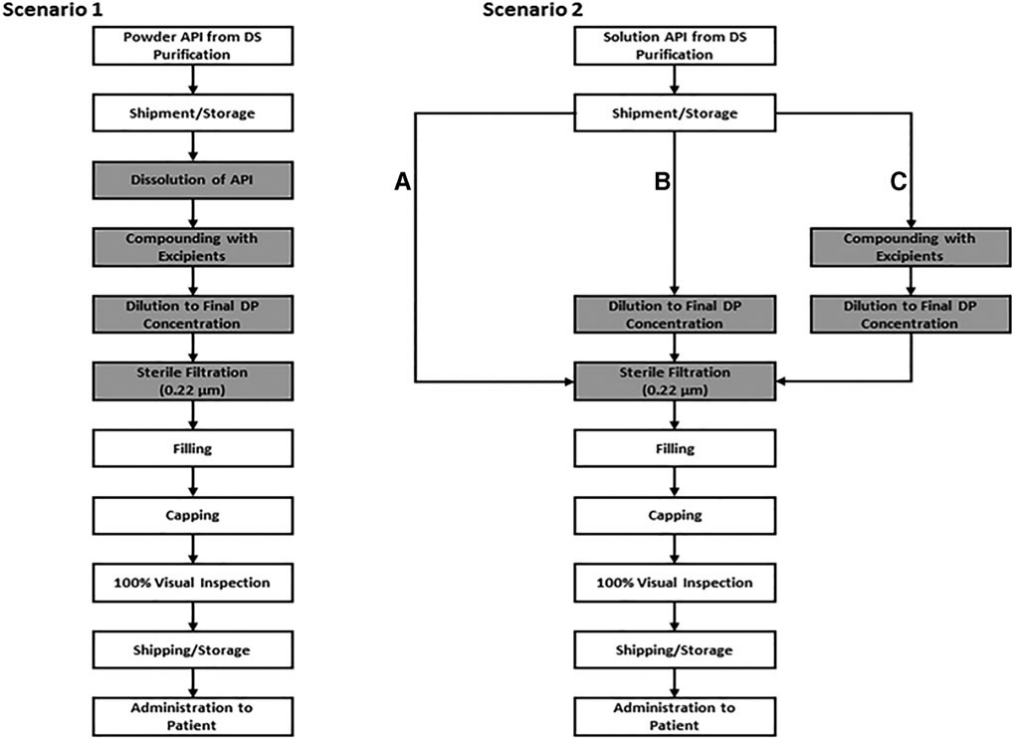
Schematic of drug product process for powder API and solution API.

One of the most attractive advantages of using a solution API is the increase in robustness and manufacturing efficiency of the DP process. A schematic for a representative DP process utilizing powder API is shown in Fig. 2 (Scenario 1). This process starts with the dissolution and compounding of the API into the formulation buffer. Compounding steps can feature the preparation of formulation buffers or the addition of excipients. Dilution with water for injection to the final DP concentration is then performed. Finally, aseptic filling is accomplished by sterile filtering the bulk DP, filling the DP into its final container closure (ie, vials, syringes), and stoppering and capping (as relevant) to provide the final configuration. Oligonucleotide DP concentrations may vary significantly depending on the method of delivery: Intravenous products generally utilize lower DP concentrations compared with products delivered subcutaneously though solubility, stability, and bioavailability generally influence the needed concentration.

The simplest case DP manufacture (Scenario 2, Path A) involves using a “ready-to-fill” solution API, being a common scenario for biologics manufacture. In this scenario, the solution API is fully formulated by compounding to the final DP concentration and adding excipients during the API manufacturing process, so the API can simply be filtered and filled into the final DP configuration. Such an approach eliminates the initial dissolution and compounding steps in the DP manufacturing process that are required with a powder API. These steps are often time-consuming and, depending on the complexity of the formulation employed, can be labor intensive with many associated manufacturing steps.

A “ready-to-fill” API may enable the most straightforward and simplest DP manufacturing process, whereas solution API can facilitate alternative scenarios when special considerations need to be applied for a particular program. For instance, in the case where several product strengths are needed to accommodate different patient populations, rather than producing a “ready-to-fill” API, a concentrated form of the API is provided in solution, that is, the API is in the final formulation but not at the final DP concentration (Scenario 2, Path B). By doing this, differences in dosage strength can be accommodated by adding a dilution step before filtration where the concentrated API is diluted with formulation buffer.

An additional scenario is to produce the API in a solution that is neither in the final formulation nor at the final concentration of the DP (Scenario 2, Path C). For example, a self-buffered solution of the API offers the advantage of the ability to manufacture with a variety of excipients and at a range of excipient and API concentrations. Such a scenario might be considered for programs at a very early stage, where the formulation is not yet fixed and a high flexibility for comparison of different formulations, for example, within formulation studies, is required.

## Conclusion

There are a number of interconnected technical considerations that must be assessed when determining whether a solution presentation is preferable to a powder for an oligonucleotide API. Adequate stability to allow for storage before DP manufacturing is a primary concern, as insufficient stability will likely preclude the use of a solution API. However, if sufficient stability is demonstrated, solution API may be considered as an alternative to powder API provided that it allows greater efficiency and flexibility for both the API and DP manufacturing processes.
